# 

**Published:** 1896-03

**Authors:** 


### BOOKS RECEIVED.

Medical Jurisprudence, Forensic Medicine and Toxicology. By R. A. Witthaus, A. M., M. D., and Tracy C. Becker, A. B., LL. B., and a staff of collaborators. In four royal 8vo volumes. Volume III. Forensic Medicine (continued). New York: William Wood & Co. 1896.

Twentieth Century Practice. An International Encyclopedia of Modern Medical Science. By leading authorities of Europe and America. Edited by Thomas L. Stedman, M. D., New York City. In twenty volumes. Volume VI.: Diseases of the Respiratory Organs. New York: William Wood & Co. 1895.

Johns Hopkins Hospital Reports. Volume V. Contents: I. Malarial Fevers of Baltimore; II. Study of Some Fatal Cases of Malaria; III. Studies in Typhoid Fever. Baltimore: The Johns Hopkins Press. 1895.

Color-Vision and Color-Blindness. A Practical Manual for Railroad Surgeons. By J. Ellis Jennings, M. D. (University of Pennsylvania), formerly Clinical Assistant Royal London Ophthalmic Hospital (Moorfields); Lecturer on Ophthalmoscopy and Chief of the Eye Clinic in the Beaumont Hospital Medical College; Ophthalmic and Aural Surgeon to the St. Louis Mullanphy and Methodist Deaconess Hospitals; Consulting Oculist to the Missouri, Kansas & Texas Railway System; Fellow of the British Laryngological and Rhinological Association. Illustrated with one colored full-page plate and twenty-one photo-engravings. Crown 8vo, 110 pages. Cloth, $1.00 net. Philadelphia: The F. A. Davis Co., Publishers. 1896.

Syphilis in the Middle Ages and in Modern Times. By Dr. F. Buret, Paris, France. Translated from the French, with notes, by A. H. Ohmann-Dumesnil, M. D., Professor of Dermatology and Syphilology in the Marion Sims College of Medicine; Consulting Dermatologist to the St. Louis City Hospital, to the St. Louis Female Hospital; Physician for Cutaneous Diseases to the Alexian Brothers' Hospital; Dermatologist to Pius Hospital, to the Rebekah Hospital, to the St. Louis Polyclinic and Emergency Hospital, etc., etc. Being Volumes II. and III. of Syphilis Today and Among the Ancients, complete in three volumes. Duodecimo, 300 pages. Extra cloth, $1.50 net. Philadelphia: The F. A. Davis Co., Publishers. 1895.

The Principles of Bacteriology. A Practical Manual for Students and Physicians. By A. C. Abbott, M. D., First Assistant, Laboratory of Hygiene, University of Pennsylvania, Philadelphia. Third edition, enlarged and thoroughly revised. Duodecimo, 492 pages, with ninety-eight illustrations, of which seventeen are colored. Cloth, $2.50. Philadelphia: Lea Brothers & Co., Publishers.

Atlas of Traumatic Fractures and Dislocations. With a Brief Treatise. By H. Helferich, M. D., Professor at the University of Greifswald. With 166 illustrations, after original drawings by Dr. Joseph Trumpp. New York: William Wood & Co. 1896.

Proceedings of the Twenty-first and Twenty-second Annual Meetings of the Oregon State Medical Society, held at Portland, May 31, June 1, 2, 1894, and June 11 and 12, 1895. Volume XX. Edited by E. F. Tucker, M. D., secretary. Portland: 1895.

Formulaire des Médications nouvelles, par le Dr. H. Gillet, ancien interne des hôpitaux de Paris, chef du service des maladies des enfants à la Polyclinique de Paris. 1 vol. in 18 de 280 pages, avec fig., cart. 3 fr. Paris: J.-B. Baillière et Fils, 19, rue Hautefeuille (près du boulevard Saint-Germain). 1896.

An Atlas of the Normal and Pathological Nervous Systems, together with a Sketch of the Anatomy, Pathology and Therapy of the same. By Dr. Christfried Jakob, practising physician in Bamberg; formerly First Assistant in the Medical Clinic at Erlangen. With an introduction by Prof. Dr. Ad. v. Strümpell. Translated and edited by Joseph Collins, M. D., Instructor of Mental and Nervous Diseases at the New

York Post-Graduate Medical School. New York: William Wood & Company. 1896.

A Manual of Medical Jurisprudence and Toxicology. By Henry C. Chapman, M. D., Professor of the Institutes of Medicine and Medical Jurisprudence in Jefferson Medical College of Philadelphia; Member of the College of Physicians of Philadelphia, etc., etc. Second edition, revised. With fifty-five illustrations and three plates in colors. Saunders' New Aid Series. Small 8vo, pp. 254. Price, $1.50. Philadelphia: W. B. Saunders. 1896.

